# Pharmacodynamics of the Orotomides against *Aspergillus fumigatus*: New Opportunities for Treatment of Multidrug-Resistant Fungal Disease

**DOI:** 10.1128/mBio.01157-17

**Published:** 2017-08-22

**Authors:** William W. Hope, Laura McEntee, Joanne Livermore, Sarah Whalley, Adam Johnson, Nicola Farrington, Ruwanthi Kolamunnage-Dona, Julie Schwartz, Anthony Kennedy, Derek Law, Michael Birch, John H. Rex

**Affiliations:** a Antimicrobial Pharmacodynamics and Therapeutics, University of Liverpool, Liverpool, United Kingdom; b Department of Biostatistics, Institute of Translational Medicine, University of Liverpool, Liverpool, United Kingdom; c Charles River Laboratories, Davis, California, USA; d F2G Ltd., Eccles, United Kingdom; Louis Stokes Veterans Affairs Medical Center

**Keywords:** *Aspergillus fumigatus*, antifungal agents, antifungal resistance, antifungal susceptibility testing, drug discovery, laboratory animals, pharmacodynamics, pharmacokinetics, pharmacology, pneumonia, pulmonary infection

## Abstract

F901318 is an antifungal agent with a novel mechanism of action and potent activity against *Aspergillus* spp. An understanding of the pharmacodynamics (PD) of F901318 is required for selection of effective regimens for study in phase II and III clinical trials. Neutropenic murine and rabbit models of invasive pulmonary aspergillosis were used. The primary PD endpoint was serum galactomannan. The relationships between drug exposure and the impacts of dose fractionation on galactomannan, survival, and histopathology were determined. The results were benchmarked against a clinically relevant exposure of posaconazole. In the murine model, administration of a total daily dose of 24 mg/kg of body weight produced consistently better responses with increasingly fractionated regimens. The ratio of the minimum total plasma concentration/MIC (*C*_min_/MIC) was the PD index that best linked drug exposure with observed effect. An average *C*_min_ (mg/liter) and *C*_min_/MIC of 0.3 and 9.1, respectively, resulted in antifungal effects equivalent to the effect of posaconazole at the upper boundary of its expected human exposures. This pattern was confirmed in a rabbit model, where *C*_min_ and *C*_min_/MIC targets of 0.1 and 3.3, respectively, produced effects previously reported for expected human exposures of isavuconazole. These targets were independent of triazole susceptibility. The pattern of maximal effect evident with these drug exposure targets was also apparent when survival and histopathological clearance were used as study endpoints. F901318 exhibits time-dependent antifungal activity. The PD targets can now be used to select regimens for phase II and III clinical trials.

## IMPORTANCE

Invasive fungal infections are common and often lethal. There are relatively few antifungal agents licensed for clinical use. Antifungal drug toxicity and the emergence of drug resistance make the treatment of these infections very challenging. F901318 is the first in a new class of antifungal agents called the orotomides. This class has a novel mechanism of action that involves the inhibition of the fungal enzyme dihydroorotate dehydrogenase. F901318 is being developed for clinical use. A deep understanding of the relationship between dosages, drug concentrations in the body, and the antifungal effect is fundamental to the identification of the regimens to administer to patients with invasive fungal infections. This study provides the necessary information to ensure that the right dose of F901318 is used the first time. Such an approach considerably reduces the risks in drug development programs and ensures that patients with few therapeutic options can receive potentially life-saving antifungal therapy at the earliest opportunity.

## INTRODUCTION

Invasive fungal diseases remain common and lethal infections in immunocompromised hosts. In clinical settings, antifungal therapy is complicated by frequent diagnostic uncertainty, difficulties in assessing the therapeutic response, antifungal drug toxicity, drug-drug interactions, and uncertainty about the appropriate duration of therapy. These well-recognized issues in the routine care of patients are now increasingly complicated by the rising specter of antifungal drug resistance (1), with mortality from azole-resistant aspergillosis of 88 to 100% in uncontrolled series (2–4). Consequently, new antifungal agents are urgently required.

The orotomides are a new series of antifungal agents with potent *in vitro* activity against *Aspergillus* spp. and other medically important molds, such as *Lemontospora* (*Scedosporium*) *prolificans*, certain species of *Fusarium*, and *Penicillium* spp., as well as *Taloromyces* (formerly *Penicillium*) *marneffei* (5). This chemical series was discovered by F2G Ltd. by screening a 300,000-compound library against *Aspergillus* spp. Notably, there is no activity of the orotomides against yeasts like *Candida* and *Cryptococcus neoformans* and no activity against the Mucorales (5). The orotomides have a completely novel mechanism of action whereby fungal pyrimidine biosynthesis is disrupted via reversible inhibition of the enzyme dihydroorotate dehydrogenase (DHODH), which is responsible for catalyzing dihydroorotate to orotate (5). Interruption of pyrimidine synthesis impairs nucleic acid production and leads to the arrest of hyphal extension. The modal MIC against *Aspergillus fumigatus* (including triazole-wild-type and -resistant strains) is 0.03 mg/liter.

Herein, we describe the pharmacodynamics of F901318 against *Aspergillus fumigatus* as a first critical step for the identification of candidate human regimens for early-phase clinical studies. We have recently considered the strategy and steps that constitute an adequate preclinical pharmacokinetic-pharmacodynamic (PK-PD) package for new antifungal agents (6) and have used this as a template for the development of F901318. A deep understanding of PK-PD relationships is required to lower the risks in clinical development programs. The use of a regimen that is too low increases the risk of concentration-dependent therapeutic failure, while an excessive dose may increase the probability of toxicity. In either case, failure to select the right regimen places the entire developmental program in jeopardy. Pharmacodynamics is a way that the risks in development programs can be reduced (6).

## RESULTS

### Murine model of IPA.

A well-characterized and severe neutropenic murine model of invasive pulmonary aspergillosis (IPA) was used (7). The severity was the same regardless of the different challenge strains of *Aspergillus fumigatus* used (the strains are summarized in Table 1). The first signs of morbidity appeared 36 to 48 h postinoculation. Mice experienced progressive weight loss throughout the experiment. Mortality began after 3 to 4 days and, without specific antifungal therapy, was nearly 100% after 7 days. A 20-mg/kg-of-body-weight dose of posaconazole per day orally was used as a positive control in all experiments. This regimen had previously been demonstrated to result in suppression of galactomannan (GM) in murine models of IPA (8).

**TABLE 1:** MICs of F901318 and posaconazole against *Aspergillus fumigatus* strains used in this study, obtained using EUCAST methodology^a^

Strain (genotype)	MIC (mode^b^) of:		Source/provenance
	Posaconazole	F901318	
NIH/4215 (triazole wild type)	0.125	0.03	National Institutes of Health, Bethesda, MD, USA
AF210 (triazole wild type)	0.125	0.03	Regional Mycology Laboratory, Manchester, UK
AF10 (triazole wild type)	0.125	0.03	Regional Mycology Laboratory, Manchester, UK
ATCC 13073 (triazole wild type)	0.125	0.03	ATCC
16216 (TR34/L98H mutant)	0.25	0.03	Regional Mycology Laboratory, Manchester, UK
11628 (G138C mutant)	0.5	0.03	Regional Mycology Laboratory, Manchester, UK
SSI6263 (TR34/L98H mutant)	2	0.015	Maiken Arendrup, Statens Serum Institut, Copenhagen, Denmark
SSI6166 (TR34/L98H mutant)	0.25	0.015	Maiken Arendrup, Statens Serum Institut, Copenhagen, Denmark

a The MICs of F901318 were the same using CLSI methodology.

b The modal (most frequent) MIC is reported.

### **Tolerability of** F901318.

Preliminary experiments suggested that the upper toxicity bound for F901318 in mice was approximately 30 mg/kg intravenously (i.v.). The administration of this dose as a rapid i.v. push into a tail vein was not tolerated. Hence, the largest dose that was used in subsequent experiments was 45 mg/kg/day administered in three divided dosages (i.e., 15 mg/kg every 8 h [q8h]). This regimen was consistently well tolerated. Higher dosages were also limited by the relative insolubility of F901318 in water and potential toxicity of the excipients used in the i.v. preparation.

### Murine pharmacokinetics.

**FIG 1:**
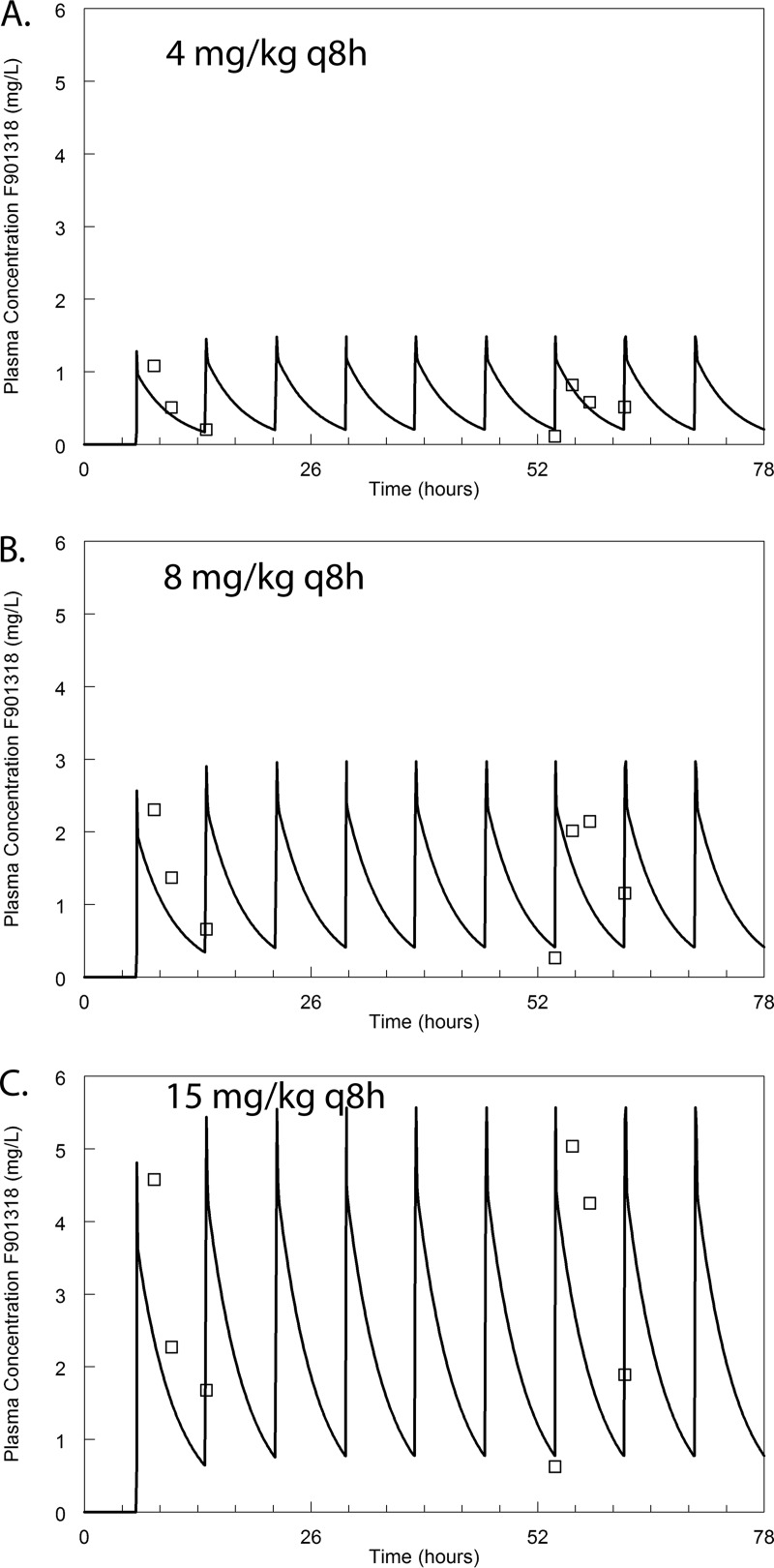
The murine plasma pharmacokinetics of F901318. The open squares represent the mean plasma concentrations obtained from groups of 3 mice. Error bars are omitted for clarity. The solid line is the fit of the population PK model to the data. The data from the first of two separate experiments are shown, although the fit of the model to the data is obtained from both experiments. The PK model fit the model well. Observed concentration = 0.266 + 0.96 × predicted concentration; *r*^*2*^ = 0.82.

The pharmacokinetics were linear in the range of 4 to 15 mg/kg q8h. These dosages were studied after preliminary dose-finding experiments and with the knowledge that they encompassed the relevant portions of the exposure-response relationship. A standard 2-compartment PK model was fitted to the data. The mean and median parameter estimates and the associated standard deviation for each model parameter are summarized in Table 2. The PK data and the fit of the pharmacokinetic model to the data are shown in Fig. 1.

**TABLE 2:** The PK parameters describing the murine plasma PK of F901318

Parameter (unit ofmeasure)^a^	Mean	Median	SD^b^	Coefficient of variation (%)
SCL (liter/h)	0.020	0.022	0.005	23.06
*V*_*c*_ (liters)	0.037	0.048	0.016	43.66
*K*_*cp*_ (h^−1^)	17.640	22.310	6.653	37.71
*K*_*pc*_ (h^−1^)	15.400	21.130	8.077	52.44

a SCL is clearance from the central compartment; *V*_*c*_ is the volume of the central compartment; *K*_*cp*_ and *K*_*pc*_ are the respective first-order intercompartmental rate constants.

b SD, standard deviation.

### Dose-fractionation studies.

**FIG 2:**
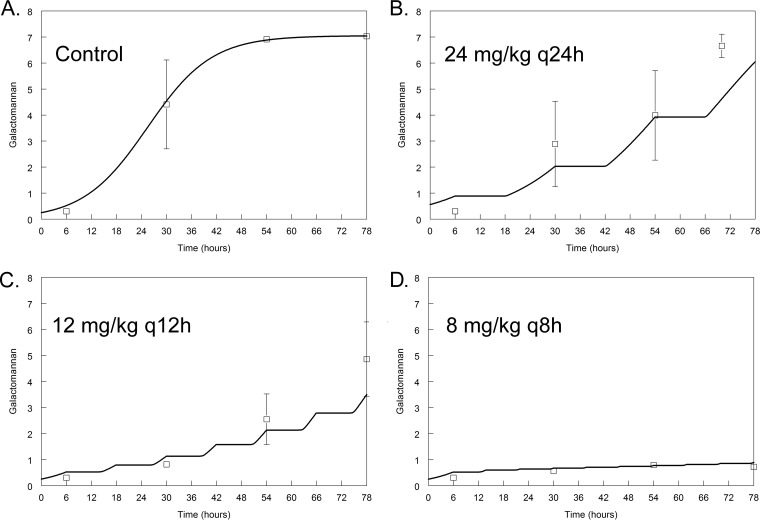
The antifungal activity of F901318 against *Aspergillus fumigatus* strain 16216, which is a TR34/L98H mutant (triazole resistant), in an isodose experiment. The open squares are the mean values ± standard errors of the means (SEM) of the galactomannan index for three mice. The solid line is the fit of the mathematical model to the PK-PD data set. In this experiment, a total daily dose of 24 mg/kg/day was administered once (B), two one-half dosages were administered twice (C), and three one-third dosages were administered every 8 h (D). There was a progressively greater effect, as smaller dosages were administered more frequently. Overall, galactomannan-time profiles were significantly different between regimens (*P* < 0.001). Compared to the results for the control, galactomannan profiles over time were significantly different for 12 mg/kg q12h (*P* = 0.028) and 8 mg/kg q8h (*P* = 0.024). No difference was detected for 24 mg/kg q24h versus the controls over time.

Isodose experiments using 24 mg/kg/day demonstrated striking differences in the antifungal effect of F901318 according to the schedule of drug administration. A regimen of 8 mg/kg F901318 administered every 8 h resulted in complete suppression of the GM levels throughout the experimental period, whereas the same total daily dosage administered q24h or q12h resulted in fungal growth (*P* < 0.001) (Fig. 2). This pattern of effect was the same regardless of the susceptibility to triazoles (see Fig. S1 in the supplemental material for dose fractionation studies for strain NIH/4215, which is triazole wild type).

10.1128/mBio.01157-17.1 FIG S1  The antifungal activity of F901318 against *Aspergillus fumigatus* strain NIH/4215 in an isodose experiment. The open squares are the mean values ± SEM from three mice. The solid line is the fit of the mathematical model to the PK-PD data set. In this experiment, a total daily dose of 24 mg/kg/day was administered once (B), two half dosages were administered twice (C) and three one-third dosages were administered every 8 h (D). There was a progressively greater effect as smaller dosages were administered more frequently. Overall, galactomannan-time profiles were significantly different between regimens (*P* = 0.0009). Download FIG S1, TIF file, 11.1 MB. Copyright © 2017 Hope et al. 2017 Hope et al. This content is distributed under the terms of the Creative Commons Attribution 4.0 International license.

During these experiments and PK-PD analyses, it was clear that the total plasma concentrations of F901318 were always greater than the MIC for both NIH/4215 and F16216 (i.e., all >0.03 mg/liter). This observation appeared inconsistent with the results of the dose fractionation experiment, where there was clear evidence of time-dependent antifungal activity (Fig. 2). Ordinarily, time-dependent antifungal activity is quantified using the fraction of the dosing interval for which either the total or free drug concentration is greater than the MIC (9). For F901318, the former was not tenable because the total plasma drug concentrations were greater than the MIC for 100% of the dosing interval. The latter was also problematic because of the high protein binding of F901318 (ca. 99% using equilibrium dialysis). Using free time (*fT*) > MIC values did not readily discriminate between the three regimens whose data are shown in Fig. 2 (i.e., the estimates for *fT* > MIC were surprisingly close for 24 mg/kg q24h, 12 mg/kg q12h, and 8 mg/kg q8h), and therefore, it could not easily account for the large differences in antifungal activity that were observed with these different schedules of drug administration. It is possible that more fractionated regimens might have enabled relationships between *fT* > MIC and effect to be established, but this was not possible with the design that was employed in this study.

**FIG 3:**
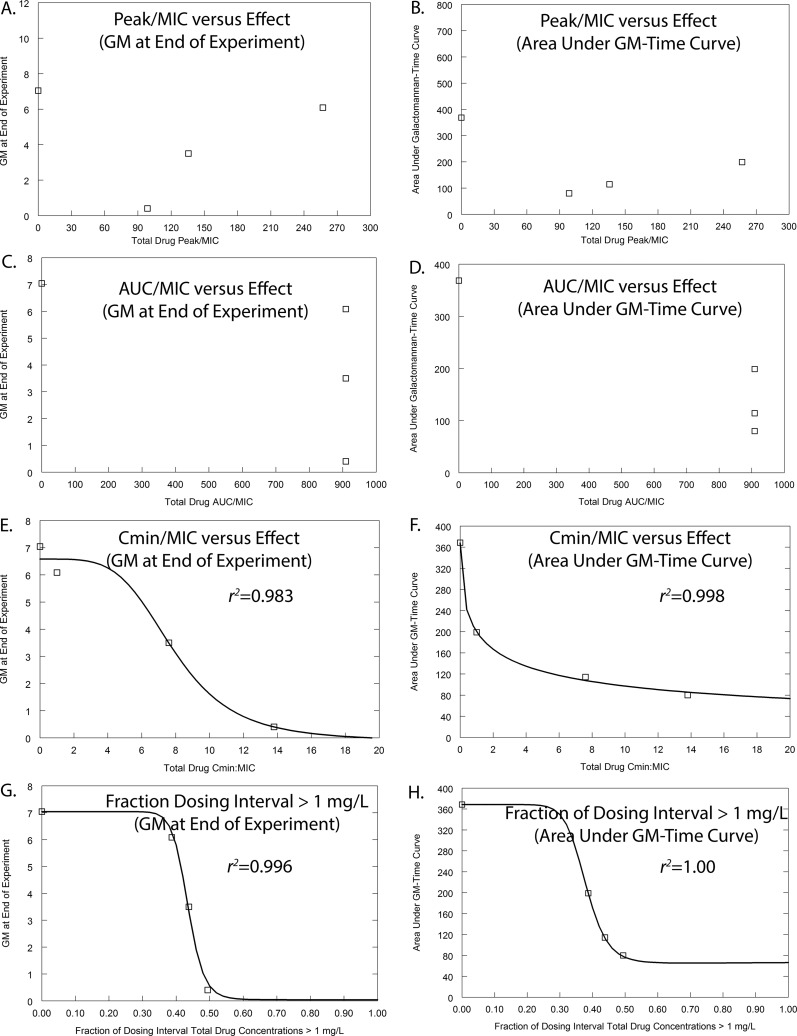
Pharmacodynamic index studies for *Aspergillus fumigatus* strain 16216, which is the same TR34/L98H mutant used in the experiment whose results are shown in Fig. 2. The pharmacodynamic data presented in Fig. 2 have been transformed from dose (mg/kg) to different pharmacodynamic indices. Two pharmacodynamic endpoints are used: the GM at the end of the experiment and the area under the GM-time curve. (A and B) The data suggest there is no relationship between *C*_max_/MIC and the observed antifungal effect. (C and D) Similarly, the data suggest there is no relationship between AUC/MIC and the observed antifungal effect. (E to H) In contrast, there is a striking relationship between the total drug *C*_min_/MIC versus effect (E and F), as well as the fractions of the dosing interval plasma concentrations that are >1 mg/liter and the antifungal effect (G and H). These analyses suggest that there is a strong threshold effect (or time-dependent) antifungal activity that is exhibited by F901318.

Alternative ways of quantifying time-dependent antifungal activity were considered, and these were as follows: (i) the total drug minimum-concentration–to–MIC ratio (*C*_min_/MIC), which is a measure of threshold effect that has been increasingly used in the resistance literature where biological activity is seen despite drug concentrations being persistently greater than the MIC (see reference 10, for example), and (ii) the fraction of the dosing interval during which plasma concentrations are greater than the MIC determined in the presence of 40% serum (this value was consistently 1 mg/liter). Thus, the two measures of the threshold effect that were used to quantify drug exposure were *C*_min_/MIC and *T* > MIC of 1 mg/liter; these are shown in Fig. 3 and also in Fig. S2.

10.1128/mBio.01157-17.2 FIG S2  Pharmacodynamic index studies for *Aspergillus fumigatus* strain NIH/4215, which is the same isolate used in the experiment whose results are shown in Fig. S1. There is no relationship between AUC/MIC and *C*_max_/MIC and the observed antifungal effect of F901318 when assessed using the area under the GM-time curve or the GM at the end of the experiment. There is a striking relationship between the total drug *C*_min_/MIC versus effect, as well as the fractions of the dosing interval plasma concentrations that are >1 mg/liter and the antifungal effect. These analyses suggest that F901318 exhibits a strong threshold effect. Download FIG S2, TIF file, 66.5 MB. Copyright © 2017 Hope et al. 2017 Hope et al. This content is distributed under the terms of the Creative Commons Attribution 4.0 International license.

### Benchmarking of the activity of F901318 against data for posaconazole.

**FIG 4:**
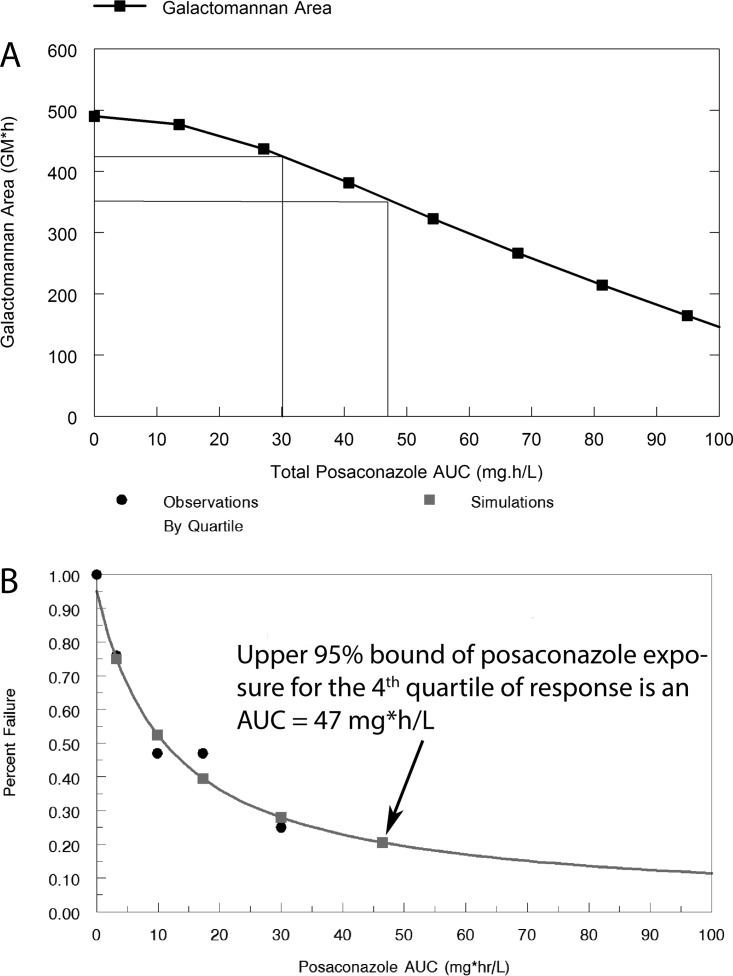
Definition of the pharmacodynamic targets used for the PK-PD bridging. (A) Relationship between posaconazole drug exposure (quantified in terms of the AUC) and the area under the galactomannan-time curve. The mean and 95% upper margin of posaconazole drug exposure (an AUC of 30 and 47 mg ⋅ h/liter, respectively) are associated with the upper-fourth quartile of the response result in 13% and 27% reductions, respectively, in galactomannan. (B) The data show the exposure-response relationship for patients receiving posaconazole as salvage therapy.

The pharmacodynamics of posaconazole was studied to better characterize the model’s behavior and aid in the interpretation of the antifungal activity of F901318. A mathematical model was fitted to the entire posaconazole data set (see below for details on the structural model and data shown in the supplemental material). The parameter values are summarized in Table S1. A posaconazole area under the concentration-time curve (AUC) of approximately 47 mg ⋅ h/liter in humans represents the upper 95% confidence bound for the upper quartile for response in patients (11). This same exposure is achieved with approximately 10 mg/kg in mice and results in a decline of 27% in the area under the GM time curve (Fig. 4).

10.1128/mBio.01157-17.3 TABLE S1  *K*_*a*_ (h^−1^) is the first-order rate constant connecting the cut with the central compartment; SCL (liter/h) is the clearance of drug from the central compartment; *V*(l) is the volume of the central compartment; *K*_*cp*_ (h^−1^) and *K*_*pc*_ (h^−1^) are the first-order intercompartmental rate constants; *K*_gmax_ (GM/h) and *k*_kill max_ (GM/h) are the maximal rates of fungal growth and drug-induced kill, respectively; Pop_max_ (GM) is the maximum theoretical fungal density; *C*_50*g*_ (mg/liter) and *C*_50*k*_ (mg/liter) are the concentrations of F901318 (or posaconazole) that induce half-maximal effects on growth and kill, respectively; and *H*_*g*_ and *H*_*k*_ are the respective slope functions for growth and kill. The initial condition (GM; not shown in the equations) is the fungal density immediately following inoculation and is estimated along with other parameters. Download TABLE S1, DOCX file, 0.1 MB. Copyright © 2017 Hope et al. 2017 Hope et al. This content is distributed under the terms of the Creative Commons Attribution 4.0 International license.

### Pharmacodynamics of F901318 against *Aspergillus fumigatus*.

**FIG 5:**
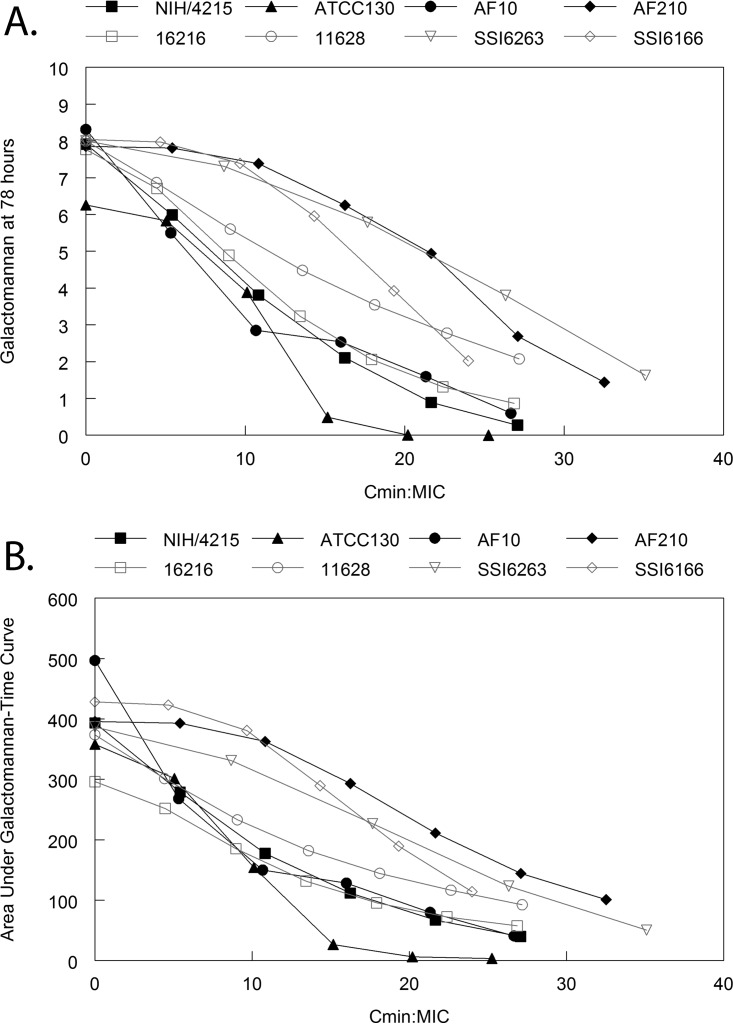
Model-predicted (i.e., from the PK-PD mathematical models fitted to the data from each strain, summarized in Table S1) relationship between drug exposure (quantified in terms of the *C*_min_/MIC) and the measured galactomannan at the end of each experiment (i.e., 78 h) (A) and area under the galactomannan-time curve from time = zero to time = 78 h (B). The black lines are the data for triazole-wild-type strains (defined phenotypically), and the grey lines are the data for triazole-resistant mutants (defined phenotypically and genotypically). As the *C*_min_/MIC increases, there is a progressively greater antifungal effect, as evidenced by lower GM levels at the end of the experiment. Using a target of 27% reduction in the area under the galactomannan-time curve (which corresponds to the effect induced by a posaconazole AUC of 47 mg ⋅ h/liter), the F901318 *C*_min_ and *C*_min_/MIC from the 8 strains were 0.27 mg/liter and 9.07, respectively. There was considerable strain-to-strain variability in the pharmacodynamics of F901318.

F901318 exposure induced a dose-dependent decline in GM of triazole-susceptible and triazole-resistant strains (Fig. 5). The exposure-response relationships were comparable whether the GM at the end of the experiment (at time = 78 h) or the area under the GM time curve (from time = 0 to time = 78 h) was used as the primary pharmacodynamic endpoint. The pharmacodynamics were comparable for triazole-susceptible and -resistant strains (Fig. 5). The *C*_min_/MIC values that achieved a 27% reduction in GM (that is, a reduction similar to that of a maximal posaconazole exposure) in the 8 challenge *Aspergillus fumigatus* strains were 3, 5, 6.5, 6.5, 7, 12.5, 15.5, and 16.5 (average *C*_min_/MIC of 9.1 and a corresponding average *C*_min_ of 0.27 mg/liter).

### Survival studies.

**FIG 6:**
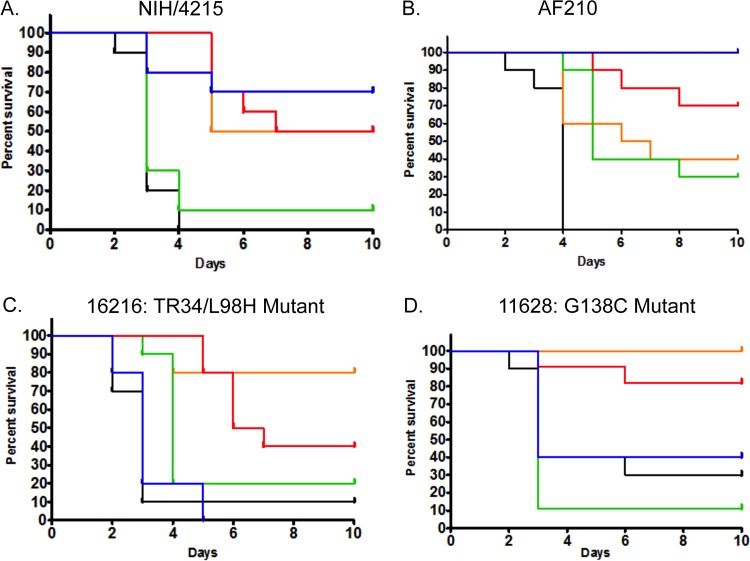
Survival of mice with IPA caused by different *A. fumigatus* strains receiving F901318 and posaconazole. Each cohort consisted of 10 mice. F901318 was administered as 24 mg/kg q24h (*C*_min_/MIC = 1.129; green), 8 mg/kg q8h (*C*_min_/MIC = 15.34; orange), and 15 mg/kg q8h (*C*_min_/MIC = 28.76; red). The impact on survival is compared with the results for vehicle controls (black) and administration of posaconazole at 10 mg/kg/day orally (blue). (A) Strain NIH/4215. Controls versus 24 mg/kg F901318 q24h, *P* = 0.396; controls versus 8 mg/kg F901318 q8h, *P* < 0.001; controls versus 15 mg/kg F901318 q8h, *P* < 0.001; controls versus 10 mg/kg posaconazole, *P* < 0.001. (B) Strain AF210. Controls versus 24 mg/kg F901318 q24h, *P* = 0.005; controls versus 8 mg/kg F901318q8h, *P* = 0.001; controls versus 15 mg/kg F901318 q8h, *P* < 0.001; controls versus 10 mg/kg posaconazole, *P* < 0.001. (C) Strain 16216 (TR34/L98H mutant). Controls versus 24 mg/kg F901318 q24h, *P* = 0.002; controls versus 8 mg/kg F901318 q8h, *P* = 0.001; controls versus 15 mg/kg F901318 q8h, *P* = 0.002; controls versus 10 mg/kg posaconazole, *P* = 0.262. (D) Strain 11628 (G138C mutant). Controls versus 24 mg/kg F901318 q24h, *P* = 0.213; controls versus 8 mg/kg F901318 q8h, *P* = 0.001; controls versus 15 mg/kg F901318 q8h, *P* = 0.004; controls versus 10 mg/kg posaconazole, *P* = 0.353. Note the reduced survival that is statistically indistinguishable from the results for the controls, for mice receiving posaconazole that were infected with a triazole-resistant strain.

Even though it was only administered for 3 days, treatment of mice with F901318 resulted in survival prolongation at 10 days compared with the survival of vehicle-treated controls. A regimen of 15 mg/kg q8h approximated the results observed with posaconazole at 10 mg/kg/day (a clinically relevant maximal drug exposure; see above) in mice infected with posaconazole-susceptible and -resistant isolates (Fig. 6). The pattern of survival prolongation induced by F901318 was the same regardless of the underlying triazole resistance mechanism. In contrast, infection with isolates with a *CYP51A* mutation that were resistant to posaconazole (Fig. 6C and D) resulted in reduced survival in mice receiving posaconazole that was not statistically different from the survival of vehicle-treated controls.

### Histopathology studies in mice.

**FIG 7:**
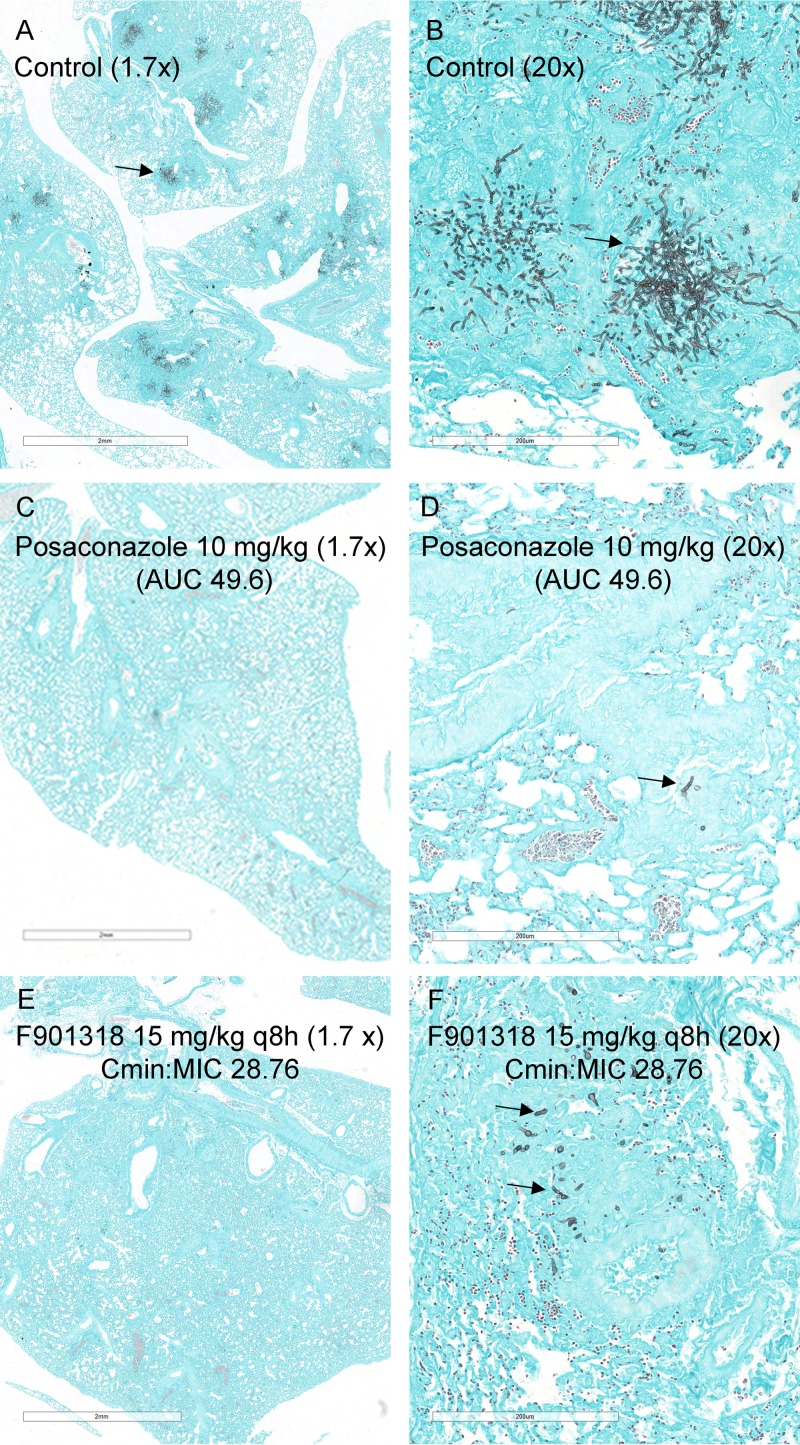
Histopathological appearances of lungs for mice infected with *Aspergillus fumigatus* NIH/4215 and treated with either posaconazole or F901318. (A) View at low-power magnification (1.7×) of lung tissue of a vehicle control mouse at 78 h postinoculation. There is multifocal pneumonia, as evidenced by multiple black patches (example highlighted by the arrow) that represent fungal invasion. (B) The patches showing multifocal pneumonia in panel A are presented at higher power (20×). (C, D) The effect of 10 mg/kg posaconazole q24h orally is shown at 1.7× (C) and 20× (D) magnification. The regimen produces a posaconazole area under the concentration-time curve (AUC) of 49.6 mg ⋅ h/liter. There is marked reduction in fungal burden compared with the results for controls. The arrow in panel D is pointing to a hypha. (E, F) The histopathological appearances for F901318 when administered at 15 mg/kg q8h are similar to those for posaconazole, which is apparent in panel E at low power (1.7×); there are occasional hyphae, shown by higher-power magnification (20×) (F) . Scale bars in panels A, C, and E, 2 mm. Scale bars in panels B, D, and F, 200 µm.

Mice were infected with NIH/4215 and treated with vehicle, posaconazole at 10 mg/kg/day orally, or F901318 at 15 mg/kg q8h (Fig. 7). In vehicle controls, there was a progressive fungal bronchopneumonia. At 6 h postinoculation, rare conidia were identified within pulmonary macrophages, within large airways, and on the luminal surface of the respiratory epithelium. There was no evidence of active inflammation or necrosis. At 30 h, there was a mild necrotizing bronchopneumonia characterized by multifocal necrosis, hemorrhage, edema, and histiocytic neutrophilic inflammation extending from the bronchi into the terminal bronchioles and alveolar space. Angioinvasion was present, with associated necrosis and inflammation of the vascular wall and/or thrombosis. At 54 h, infection was fulminant and characterized by severe inflammation, necrosis, hemorrhage, edema, necrotizing vasculitis, and vascular invasion. The histopathological findings at 78 h were similar in character and severity to those identified at 54 h.

In mice treated with 10 mg/kg posaconazole, the fungal density was reduced compared with that in controls and hyphae were contained within areas of pyogranulomatous inflammation. Consistent with its lack of effect on GM, a dose of F901318 at 24 mg/kg q24 had a minimal effect compared with that in controls. The use of a higher dose of 15 mg/kg q8h resulted in appearances similar to those in mice receiving posaconazole. However, minimal or mild pulmonary hemorrhage, edema, and/or vascular thrombosis persisted in this dose group, suggesting some unresolved vascular damage. Hyphae were contained by pyogranulomatous inflammation.

### Rabbit model of invasive pulmonary aspergillosis.

The oral administration of F901318 induced dose-dependent declines in serum GM in rabbits. In 6 of 18 rabbits, the baseline GM (i.e., GM prior to inoculation of conidia) was strongly positive (>3) and did not fluctuate throughout the experimental period, suggesting that readings were falsely positive. The pharmacodynamic data from these rabbits were excluded (because they was uninterpretable), although the pharmacokinetic data were available and used for PK-PD modeling.

**FIG 8:**
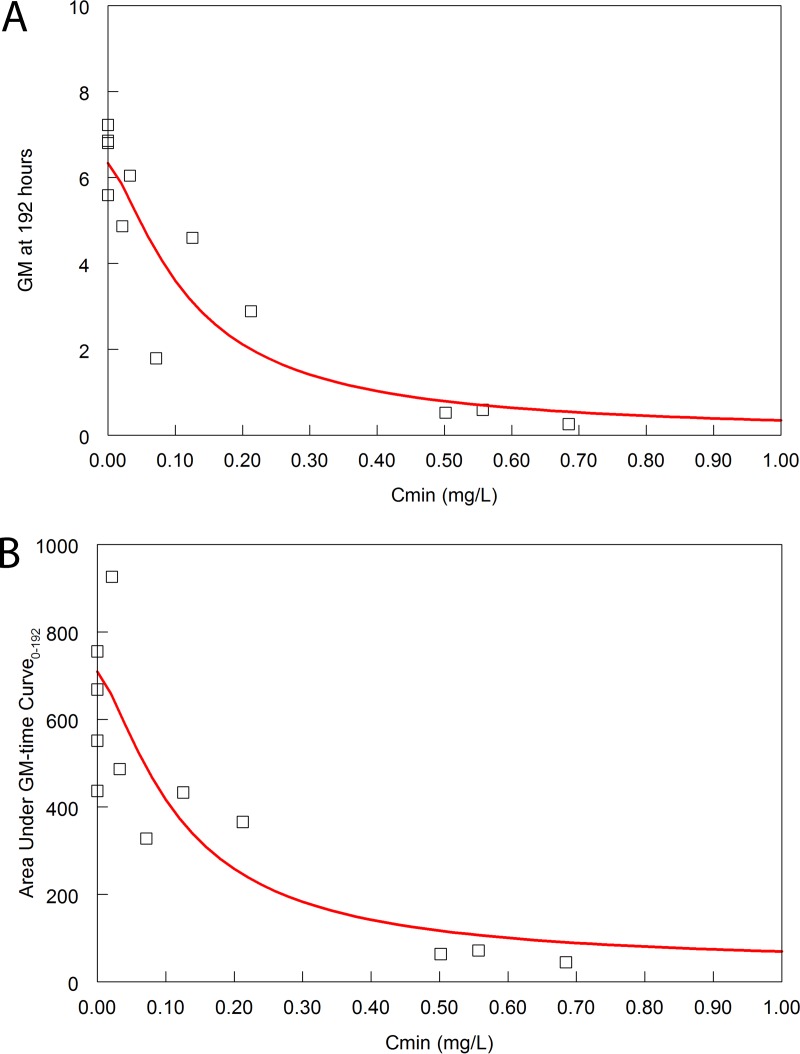
(A) Terminal GM versus *C*_min_. The red line is the fit of the sigmoid *E*_max_ model to the data. Each open square is the GM at the end of the experiment (time = 192 h postinoculation) for each of the 12 rabbits for which pharmacodynamic data were available. The sigmoid *E*_max_ model is given by the following equation: GM = 6.62 − [(6.50 × *C*_min_^1.40^)/(0.12^1.40^ + *C*_min_^1.40^)]; *r*^*2*^ = 0.84. (B) Area under the GM-time curve versus *C*_min_. The red line is the fit of the sigmoid *E*_max_ model to the data. Each open square is the area under the GM-time curve for each of the 12 rabbits for which pharmacodynamic data was available. The sigmoid *E*_max_ model is given by the following equation: area under the GM-time curve = 708.9 − [(672.5 × *C*_min_^1.23^)/(0.1^1^.^23^+ *C*_min_^1.23^)]; *r*^*2*^ = 0.73. The *C*_min_ values and the area under the GM-time curve for each rabbit were calculated from the Bayesian posterior estimates that were obtained from the linked PK-PD mathematical model.

The mathematical model fit the PK-PD data well. The parameter estimates are summarized in Table S1. The mathematical model was used to calculate the *C*_min_ value, the area under the GM time curve, and the GM at the end of the experimental period for each rabbit. The relationships between *C*_min_ (as the relevant pharmacodynamic measure of drug exposure defined in mice) and the two measures of pharmacodynamic response are shown in Fig. 8. Half-maximal activity was evident, with a *C*_min_ of 0.1 mg/liter and a corresponding *C*_min_/MIC of 3.33. In this rabbit model, a half-maximal reduction in GM has been shown to be produced by the mean steady-state exposure of isavuconazole (12).

## DISCUSSION

New antifungal agents are urgently required for clinical use. There are simply too few agents to circumvent many of the obstacles that are encountered in routine clinical care. As is the case with many anti-*Aspergillus* compounds, F901318 is relatively insoluble in water and highly protein bound (ca. 99%). Nevertheless, there are several highly attractive features that suggest this new compound can address an unmet medical need: it is orally bioavailable, active against triazole-resistant fungi, and has potent *in vitro* activity against rare neglected fungal pathogens. The principal purpose of this preclinical PK-PD study was to provide a rigorous justification of the antifungal regimens for the very first patients with invasive fungal infections. Such an approach is new for antifungal therapeutics. It reduces guesswork, prevents targeting an arbitrarily chosen plasma concentration value like the MIC, and guides decision-making in early-clinical-phase development programs.

The orotomides are the first new class of antifungal agent to be discovered for 3 decades. They were initially discovered by screening a large compound library against *Aspergillus fumigatus*, rather than the more commonly used *Candida albicans*. An understanding of the mechanism of action was established via a combination of microbiological, genetic, and biochemical approaches that are described in detail elsewhere (5). Briefly, the principal strategy was to identify genes that when present in multiple copies confer resistance to F901318. Extra copies of the gene *pyrE*, which encodes dihydroorotate dehydrogenase (DHODH), confer resistance to F901318. Furthermore, disruption of this gene returned the *in vitro* susceptibility to wild-type levels. F901318 inhibits human DHODH but is approximately <2,200-fold less active against the human enzyme than the fungal one. As expected, there is no evidence of cross-resistance with other commonly encountered antifungal resistance mechanisms, such as mutations in the Cyp51 protein. More surprising, perhaps, is the observation that resistance is not induced in the laboratory by serial passage in the presence of the drug (5). These features suggest that F901318 has the potential to be a useful agent for the treatment of patients with invasive aspergillosis caused by triazole-resistant *Aspergillus* spp.

Antifungal pharmacodynamics is a new and rapidly developing field of study. The past 5 to 10 years have seen considerable progress in terms of developing new experimental platforms and gaining a more detailed understanding of the behavior of currently available compounds in those models. In this study, preclinical PK-PD models of invasive pulmonary aspergillosis provide the underpinning evidence that is required to identify candidate regimens (i.e., both the dosage and the schedule of administration) that are likely to be associated with maximal antifungal activity in the clinic. F901318 demonstrates time-dependent antifungal activity. Regimens that result in trough concentration (*C*_min_)-MIC ratios of ∼10 result in a decline in GM that is greater than observed with clinically equivalent serum drug exposures of posaconazole. Given that the modal MIC of F901318 to the strains of *Aspergillus fumigatus* used in this study was 0.03 mg/liter, this is equivalent to a total drug *C*_min_ of approximately 0.3 mg/liter. The rabbit model suggests a lower target, with half-maximal antifungal activity evident with a *C*_min_ of 0.1 mg/liter. Collectively, therefore, *C*_min_ values of 0.1 to 0.3 are reasonable and can be used to design human regimens that are safe and effective. If well tolerated, the use of higher targets may result in additional antifungal activity. These target values are predicted to result in antifungal activity that matches or exceeds that induced by other licensed agents for invasive aspergillosis, such as posaconazole, isavuconazole, and liposomal amphotericin B (8, 12, 13).

The clinical development of F901318 is challenging. There is no possibility of using the traditional and extensively utilized pathway for the development of antifungal agents, which begins with studies in esophageal candidiasis before proceeding to clinical trials in invasive candidiasis and salvage studies in invasive aspergillosis (see, for example, the clinical development pathway followed for all three echinocandins [14–16]). Salvage studies are difficult to interpret. Furthermore, the administration of a new antifungal drug on a backbone with a known active antifungal compound presents a series of challenges without entirely satisfactory solutions. Hence, new ways of identifying clinical regimens for which the risks are reduced as much as possible for the very first patients with invasive aspergillosis receiving a new antifungal agent are required. In this regard, pharmacodynamics has a vital role to play. Antifungal pharmacodynamics is a relatively young discipline but has already made significant contributions to the setting of *in vitro* susceptibility breakpoints (8, 17), target selection for therapeutic drug monitoring (TDM) (18, 19), and dosage selection in special populations (20, 21).

This study has highlighted several issues that are pertinent for the application of modern pharmacodynamics for antifungal drug development. Some of these issues have been previously summarized by us (6). First, the importance of experimental models that are faithful mimics of human disease cannot be overstated. The murine and rabbit models in this study are both models of invasive pulmonary aspergillosis, which is the most clinically relevant manifestation of human disease, where >90% of cases involve the lung (22). The use of two experimental models minimizes the chance of an aberrant result due to species-specific idiosyncrasies in pharmacokinetics and pharmacodynamics. Second, we focused intently on establishing the study endpoints that are potentially clinically relevant by benchmarking against licensed comparators. Third, we used both wild-type strains and strains with reduced susceptibility to triazoles to demonstrate that the pharmacodynamics of F901318 is not affected by the underlying triazole resistance mechanism.

In conclusion, F901318 is a new antifungal agent with activity against *Aspergillus fumigatus*, including strains that are resistant to the triazoles. A detailed understanding of the pharmacodynamics of F901318 provides the underpinning evidence and rationale to enable the further study of this compound for patients with life-threatening invasive fungal infections with few or no other therapeutic options.

## MATERIALS AND METHODS

### Challenge strain(s) of *Aspergillus fumigatus*.

The pharmacodynamics of F901318 was determined using eight strains of *Aspergillus fumigatus*. The provenance of these strains is summarized in Table 1. Strains were triazole wild type (as defined by *in vitro* susceptibility testing; *n* = 4) and triazole resistant (as defined by *in vitro* susceptibility testing and sequencing of the Cyp51A gene; *n* = 4). MICs were determined over the course of multiple independently conducted experiments using both EUCAST and CLSI methodology.

For all strains, *Aspergillus fumigatus* conidia were prepared by subculturing on Sabouraud dextrose agar with chloramphenicol (Sigma-Aldrich, United Kingdom). Plates were incubated at 37°C for at least 5 days. To harvest conidia, the surface of the flask was gently irrigated with phosphate-buffered saline (PBS) with 0.05% Tween 80 and the resultant mixture centrifuged. The pellet was resuspended in PBS with 0.05% Tween 80. Conidia were washed through gauze, and the suspension progressively diluted to achieve a density of 1 × 10^7^ CFU/ml. The suspension was used immediately for inoculation.

### Murine model of invasive pulmonary aspergillosis.

Male CD1 mice were purchased from Charles River, Inc., and rested for 1 week prior to experimentation. Mice were housed in individually vented cages (IVCs) with 3 animals per cage and weighed 25 to 30 g at the time of experimentation. Food and water were provided *ad libitum*. All experiments were conducted under UK Home Office license PPL 40/3630 and approved by the Animal Welfare and Ethical Review Body of the University of Liverpool.

Mice were rendered neutropenic with intraperitoneal injection of 150 mg/kg cyclophosphamide on day −4 and 100 mg/kg on day −1 relative to the time of infection. Mice were further immunosuppressed with cortisone acetate (250 mg/kg) given subcutaneously on day −1 relative to the time of infection to impair the function of pulmonary alveolar macrophages.

The fungal inoculum was prepared as described above. Mice were anesthetized with 2% isoflurane. A conidial suspension of 5 × 10^5^ CFU/ml (50 µl/mouse of the 1 × 10^7^ CFU/ml inoculum) was instilled in both nares. Treatment with antifungal agents was delayed for 6 h postinoculation. Mice were treated throughout the experimental period with vehicle control (i.v.), various regimens of F901318 (i.v.), or posaconazole at 10 or 20 mg/kg orally. The treatment duration was 72 h, meaning that the entire experimental duration was 78 h (6-h treatment delay followed by 72 h of antifungal therapy).

### Study drugs.

F901318 for i.v. injection was prepared in the following way. The desired amount of drug was weighed, added to dimethyl sulfoxide (DMSO), and vortexed until fully dissolved. Subsequently, polyethylene glycol 400 (PEG 400) was added and the mixture vortexed. A solution of 35.3% hydroxyl propyl β cyclodextrin (HPBCD) was prepared in water. The solution of F901318 in DMSO-PEG and the HPBCD solution were mixed to give a clear solution. The final excipient concentrations were 30% HPCD, 5% DMSO, and 10% PEG 400. Appropriate volumes of F901318 were prepared and stored at −20°C for daily use. Prior to use, drug-containing vials were fully thawed and vortexed. An oral suspension of posaconazole (Noxafil; Merck, Inc., USA) was used and diluted with 20% HPBCD (4 g HPBCD in 20 ml water) as previously described (8).

### Measurement of F901318.

The F901318 concentrations in murine plasma were measured using high-performance liquid chromatography (HPLC) with a Shimadzu Prominence instrument (Shimadzu, Milton Keynes, United Kingdom). The F901318 method used a 50- by 2.0-mm Synergi 4-μm Max RP 80A column (Phenomenex, Macclesfield, United Kingdom) and a 40-µl injection volume. A standard curve encompassing 0.01 to 20 mg/liter was constructed from stock solutions of F901318 at 1,000 mg/liter in DMSO that were further diluted in methanol (Fisher Scientific, Loughborough, United Kingdom).

The internal standard was F901351 (F2G, Eccles, United Kingdom), which is a member of the orotomide antifungal class. Chromatographic separation was achieved using a gradient with the starting condition of 80:20 (0.1% trifluoroacetic acid [TFA] in water as mobile phase A and 0.1% TFA in acetonitrile as mobile phase B). Mobile phase B was increased to 50% over 3 min and to 65% B over minutes 3 to 5 and then reduced to the starting condition for 2.5 min of equilibration, all at a flow rate of 1 ml/min.

F901318 and F901351 were detected using UV detection with 250-nm and 270-nm wavelengths; they eluted after 4.9 and 1.9 min, respectively. The coefficient of variation (CV%) was <12.4% over the concentration range of 0.01 to 20 mg/liter. The limit of detection was 0.01 mg/liter. The intra- and interday variation was <8.2%. The assay was separately validated for rabbit plasma. The CV% was <10.4% over the concentration range of 0.05 to 20 mg/liter. The limit of detection was 0.05 mg/liter. The intra- and interday variation was <9.1%.

### Pharmacokinetics.

The plasma PK of F901318 was determined in two independently conducted experiments. Plasma drug concentrations were measured as described above. Three regimens were studied (with prior knowledge that these regimens encompassed relevant drug exposures at which antifungal activity was observed; see “Pharmacodynamics,” below). Regimens of 4 mg/kg q8h, 8 mg/kg q8h, and 15 mg/kg q8h i.v. were studied. Sampling was performed in the first dose interval from 0 to 8 h and then later between 54 and 62 h. Plasma was collected at 2, 4, and 8 h postdose, with the predose concentration being collected at 54 h. Three mice were studied at every dosage time point. Mice were terminally anesthetized using 2% isoflurane. Blood was obtained via cardiac puncture, placed in heparinized tubes, and immediately placed on ice before being centrifuged. The plasma was removed and stored at −80°C until future analysis.

### Dose fractionation studies and elucidation of the pharmacodynamic index best linking drug exposure with antifungal effect.

Three experimental pieces of evidence were used to elucidate the relevant pharmacodynamic index: (i) dose fractionation studies using triazole-wild-type strain NIH/4215 as the challenge strain and GM as the experimental read out; (ii) dose fractionation studies using an *Aspergillus fumigatus* TR34/L98H mutant (bearing a change of L to H at position 98) as the challenge strain and GM as the experimental read out; and (iii) a series of survival studies using strains NIH/4215 (triazole wild type), AF210 (triazole wild type), 16216 (a TR34/L98H mutant that has reduced susceptibility to triazoles), and 11628 (a G138C mutant [bearing a change of G to C at position 138] that is triazole resistant). In both mutants, the mutation affects the Cyp51A protein, which is the common target site for the triazole antifungal class.

The mathematical PK-PD model was used to transform drug exposure quantified in terms of the mouse (i.e., mg/kg) to measures made in terms of the invading fungal pathogen in the mouse (i.e., AUC, peak concentration, and fraction of the dosing interval plasma concentration were above threshold). These measures were then related to the MIC of the invading pathogen to generate AUC/MIC, *C*_max_/MIC, and *T* > MIC ratios. The antifungal effect of F901318 was measured by estimating both the area under the GM time curve and the GM at the end of the experiment. Different measures of drug exposure of F901318 (i.e., AUC/MIC, peak concentration/MIC, and fraction of the dosing interval drug concentration were greater than the MIC) were determined, and any possible relationship with both the area under the GM-time curve and the GM at the end of the experiment was explored by fitting inhibitory sigmoid maximum effect (*E*_max_) models to the data using nonlinear regression and the ADAPT 5 program (23).

The relevant pharmacodynamic index was assessed using dose fractionation studies in which a total daily dose of F901318 was administered as a single full dose administered once per day, two one-half dosages every 12 h, and three one-third dosages every 8 h. Preliminary dose-finding experiments were used to define the relevant areas of the exposure-response relationship where PD relationships could be elucidated. This therapeutic window proved to be relatively narrow. The administration of regimens comprising <24 mg/kg/day resulted in submaximal antifungal activity, whereas the administration of >24 mg/kg/day resulted in nearly maximal antifungal activity. In either case, the different antifungal activities of the various regimens could not be demonstrated. Hence, a single total daily dosage of 24 mg/kg/day was used for these experiments. The three fractionated regimens were 24 mg/kg/day q24h, 12 mg/kg/day q12h, and 8 mg/kg q8h (all administered intravenously). A regression model was fitted to the data with the time × drug interaction term to determine the effects of different schedules of administration on the antifungal effect.

Three experimental pieces were used to elucidate the relevant PD index: (i) dose fractionation studies using triazole-wild-type NIH/4215 as the challenge strain and GM as the experimental read out; (ii) dose fractionation studies using an *Aspergillus fumigatus* TR34/L98H mutant as the challenge strain and GM as the experimental read out; and (iii) a series of survival studies using NIH/4215 (triazole wild type), AF210 (triazole wild type), 16216 (a TR34/L98H mutant that is triazole resistant), and 11628 (a G138C mutant that is triazole resistant).

### Benchmarking against posaconazole.

As with any model, the experimental conditions used for the murine model of IPA were designed to produce “on-scale” readouts following antifungal therapy (i.e., clinically relevant drug exposures produce effects somewhere between none and maximal). The experimental conditions are such that not all regimens exert maximal antifungal effect or produce nothing at all. The challenge strain, inoculum, immunosuppression, and time to initiation of antifungal therapy all have an impact on the observed exposure-response relationships. Thus, the potential clinical significance of a decline of a biomarker induced by a new antifungal agent must be placed in some clinically relevant context. We used a previous study that examined the pharmacodynamics for posaconazole for IPA to calibrate the model’s behavior (8).

### Exposure-response relationships for F901318 and magnitude of the pharmacodynamic target associated with maximal antifungal activity.

The pharmacodynamics of F901318 against various strains of *Aspergillus fumigatus* (Table 1) was determined over the course of multiple independently conducted experiments. The same neutropenic model of IPA described above was used for each strain. A variety of regimens informed by preliminary dose-finding studies were used in these experiments. All experiments included a positive-control cohort of mice that received 20 mg/kg/day posaconazole by gavage. Such a regimen results in drug exposure that is significantly greater than that achievable in the clinic and routinely resulted in completely suppressed GM profiles.

### Survival studies.

Two triazole-wild-type strains (NIH/4215 and AF210) and two triazole-resistant strains were studied (strain 16216, a TR34/mutant, and strain 11628, a G138C mutant). Details of these strains are provided in Table 1. The neutropenic murine model of IPA described above was used but was adapted for survival studies. Each cohort consisted of 10 mice. F901318 was administered as 24 mg q24h, 8 mg/kg q8h, and 15 mg/kg q8h i.v. for 3 days, after which treatment was stopped and mice were observed. Posaconazole at 10 mg/kg/day orally was used as a comparator for these experiments. At this dose, posaconazole produces an AUC from 0 to 24 h (AUC_0−24_) of ∼50 mg ⋅ h/liter in mice, which is just above the upper 95% confidence interval for the fourth quartile of clinical response for patients with invasive aspergillosis receiving posaconazole as salvage therapy (11). For reference, the average exposures in the upper quartile of response are approximately 30 mg ⋅ h/liter. Mice were sacrificed if they reached predetermined endpoints that suggested death was imminent.

### Histopathology studies.

Sections of lung were fixed in 10% formaldehyde and stained with hematoxylin and eosin (HE) or Grocott-Gomori methenamine silver (GMS) stain.

### Pharmacokinetic and pharmacodynamic mathematical modeling.

Several PK-PD models were fitted to the data. For the PK data considered in isolation, a two-compartment PK model best described the PK data, with time-delimited zero-order input and first-order elimination from the central compartment. All models were fitted to the data using the population pharmacokinetic program Pmetrics (24). In these analyses, an “individual” consisted of a cohort of mice receiving a given regimen.

The combined PK-PD datasets (i.e., concentrations of F901318 and corresponding GM data) were modeled using a linked PK-PD mathematical model, which took the following form:

(1) $$XP\left(1\right)=R\left(1\right)-\left(\frac{\text{SCL}}{V}\right)\cdot X(1)-K_{cp}\cdot X\left(1\right)+K_{pc}\cdot X(2)$$

(2) $$XP\left(2\right)=K_{cp}\cdot X\left(1\right)-K_{pc}\cdot X(2)$$

(3) $$XP\left(3\right)=K_{g\text{ max}}\cdot \left(1-\left\{\frac{{\left[\frac{X\left(1\right)}{V}\right]}^{H_{g}}}{{C_{50g}}^{H_{g}}+{\left[\frac{X\left(1\right)}{V}\right]}^{H_{g}}}\right\}\right)\cdot X\left(3\right)\cdot \left\{1-\left[\frac{X\left(3\right)}{{\text{Pop}}_{\max}}\right]\right\}-k_{\text{kill max}}\cdot \left\{\frac{{\left[\frac{X\left(1\right)}{V}\right]}^{H_{k}}}{{C_{50k}}^{{H_{k}}^{}}+{\left[\frac{X\left(1\right)}{V}\right]}^{H_{k}}}\right\}\cdot X{\left(3\right)}^{}$$

and had output equations *Y*(1) = *X*(1)/*V* and *Y*(2) = *X*(3). *X* is the amount of drug in a compartment, *R*(1) represents the i.v. injection of F901318, SCL (liter/h) is the clearance of drug from the central compartment, and *V*(liter) is the volume of the central compartment. *K_cp_* (h^−1^) and *K_pc_* (h^−1^) are the first-order intercompartmental rate constants connecting the central (*c*) and peripheral (*p*) compartments, and *K*_gmax_ (GM/h) and *k*_kill max_ (GM/h) are the maximal rates of fungal growth and drug-induced kill, respectively. Pop_max_ (GM) is the maximum theoretical fungal density, *C*_50*g*_ (mg/liter) and *C*_50*k*_ (mg/liter) are the concentrations of F901318 (or posaconazole) that induce half-maximal effects on growth and kill, respectively, and *H_g_* and *H_k_* are the respective slope functions for growth and kill. The initial condition (GM; not shown in the equations) is the fungal density immediately following inoculation and is estimated along with other parameters.

Equations 1 and 2 are standard pharmacokinetic equations that describe a 2-compartment PK model with first-order clearance of drug from the central compartment. The pharmacodynamics of F901318 (or posaconazole) against *Aspergillus fumigatus* is described by equation 3, which has terms that describe the capacity-limited growth of *Aspergillus*, drug-induced suppression of growth, and drug-induced fungal killing. The first output equation provides the concentration-time profile of F901318, while the second provides the time course of GM. Both the pharmacokinetic and pharmacodynamic profiles can be integrated to provide the area under the concentration-time and GM time curves. For posaconazole, an additional equation describing the absorption of drug from the gut following oral administration was used.

The model was fitted to the data using Pmetrics (24). An “individual” consisted of a cohort of mice with the same experimental conditions.

### Rabbit model of IPA.

The pharmacodynamics of F901318 were further studied in a well-characterized neutropenic rabbit model of invasive pulmonary aspergillosis. This model has been used to study most of the currently licensed antifungal agents, including posaconazole, isavuconazole, and various formulations of amphotericin B (12, 13, 25).

Rabbits received F901318 by oral gavage using a spray-dried dispersion. The dosing formulation was prepared as follows. Prepared component A was 0.552 g sodium dihydrogen phosphate monohydrate (molecular weight [MW] = 137.99) in 100 g ultrahigh purity (UHP) water. Prepared component B was 1.072 g disodium hydrogen phosphate heptahydrate (MW = 268.03) in 100 g UHP water. Forty-millimolar phosphate buffer was made by mixing 23.6 g of component A and 76.4 g of component B. The pH of the buffer was monitored and adjusted with each component as necessary until a pH of 7.4 ± 0.1 was achieved. Foil pouches containing 10% F901318 and 90% hypromellose acetate succinate (HPMCAS) were stored at 4°C and allowed to reach room temperature before opening. The desired amount of drug was weighed out (with F901318 comprising 10% of the weight of the powder), added to the required volume of 40 mM phosphate buffer, and vortexed until dissolved. The F901318 oral formulation was mixed before dosing and used within an hour of preparation.

A Silastic intravenous indwelling catheter was placed in each animal. Rabbits were immunosuppressed with 525 mg/m^2^ cytosine arabinoside on day −1 relative to inoculation, and 5 mg/kg methylprednisolone was administered on day −1 and day zero. Opportunistic systemic bacterial infections were prevented with 15 mg/kg/day vancomycin, 5 mg/kg gentamicin every second day, and 75 mg/kg ceftazidime twice daily. Rabbits were inoculated via the endobronchial instillation of 1 × 10^8^ conidia from *Aspergillus fumigatus* NIH/4215 in 0.25 ml. The model read out was the serum concentration of GM as previously described in this model (13, 25, 26).

Rabbits received F901318 (0.5, 2.5, 10, and 20 mg/kg q12h) or vehicle control by oral gavage. These regimens were designed after preliminary tolerability and pharmacokinetic studies (data not shown). The ensuing drug exposure was assessed in the context of the conclusions of the murine studies described above. There were four animals per group. Treatment started 24 h postinoculation and continued for 7 days before all rabbits were sacrificed via the i.v. injection of pentobarbitone. (i.e., 192 h postinoculation.) Serum GM concentrations were determined on days 0, 1, 2, 4, 6, 7, and 8 in study 1 and days −1, 0, 1, 3, 5, 7, and 8 in study 2. The pharmacokinetics of F901318 was estimated after the first dosing interval on days 1 and 8.

### Data analysis and mathematical modeling of PK-PD data from the rabbit model.

The same structural mathematical model described for mice was used for rabbits, except that an absorptive compartment was added to enable the transit of drug from the gut to the bloodstream to be described. Each rabbit was treated as an individual in this analysis. The Bayesian estimates for the PK-PD parameters from each rabbit were obtained and used to estimate the *C*_min_ and both the area under the GM time curve for each rabbit and the GM at the end of the experiment. The relationship between drug exposure and the decline in the biomarker was then described using an inhibitory sigmoid *E*_max_ model.
