# Supplementary figures and images for: Pharmacodynamics of the Orotomides against Aspergillus fumigatus: New Opportunities for Treatment of Multidrug-Resistant Fungal Disease
Source: mBio. 2017 Aug 22;8(4):e01157-17. doi: 10.1128/mBio.01157-17 (PMC5565967; doi:10.1128/mBio.01157-17)

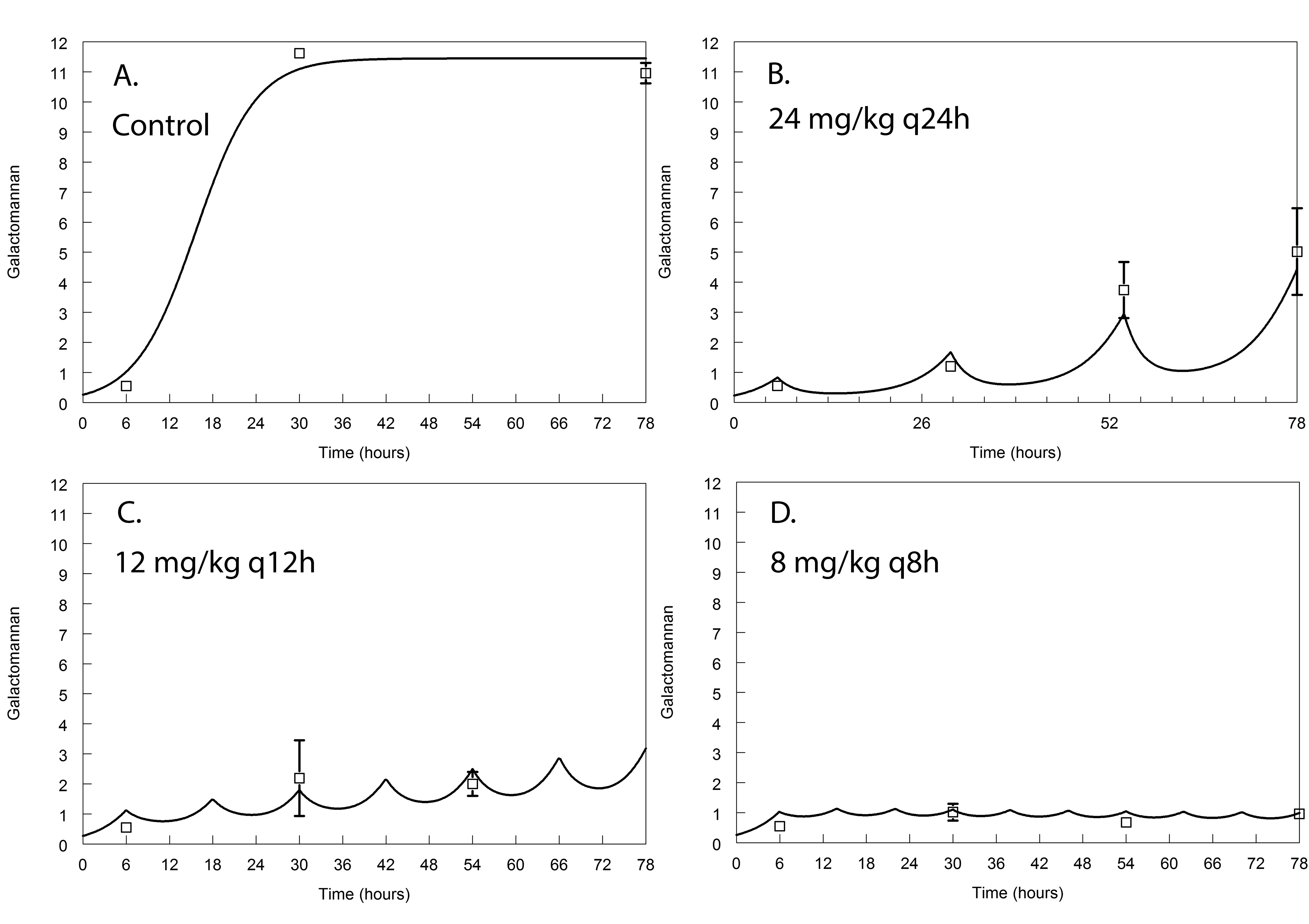

Supplement: FIG S1 [file mbo004173435sf1.tif]
