# Supplementary material for: Pharmacodynamics of the Orotomides against Aspergillus fumigatus: New Opportunities for Treatment of Multidrug-Resistant Fungal Disease
Source: mBio. 2017 Aug 22;8(4):e01157-17. doi: 10.1128/mBio.01157-17 (PMC5565967; doi:10.1128/mBio.01157-17)
Supplement: TABLE S1 [file mbo004173435st1.docx]

Supplementary Table 1. Summary of fits of various models to the data

| Model | NIH/4215 | AF210 | AF10 | ATCC13073 | 16216 | 11628 | SSI6263 | SSI6166 | Posaconazole | F901318 rabbit |
| --- | --- | --- | --- | --- | --- | --- | --- | --- | --- | --- |
| Ka (h^-1^) |  |  |  |  |  |  |  |  | 20.76 | 6.81 |
| SCL (L/h) | 0.02 | 0.02 | 0.02 | 0.02 | 0.02 | 0.02 | 0.02 | 0.02 | 0.0046 | 2.52 |
| Vc (L) | 0.05 | 0.06 | 0.09 | 0.06 | 0.07 | 0.06 | 0.05 | 0.06 | 0.05 | 18.72 |
| Kcp (h^-1^) | 12.31 | 13.60 | 1.78 | 12.68 | 6.80 | 6.70 | 15.34 | 10.93 | 10.38 | 23.42 |
| Kpc (h^-1^) | 23.93 | 19.72 | 19.36 | 20.70 | 23.42 | 14.89 | 20.92 | 22.97 | 25.51 | 11.75 |
| Kgmax (GM/h) | 0.09 | 0.08 | 0.22 | 0.20 | 0.06 | 0.10 | 0.17 | 0.10 | 0.35 | 0.11 |
| Hg | 12.01 | 3.81 | 9.26 | 0.96 | 19.98 | 12.69 | 19.85 | 15.55 | 6.36 | 10.68 |
| C50g (mg/L) | 4.36 | 2.36 | 2.65 | 2.58 | 7.96 | 5.98 | 6.36 | 7.73 | 7.85 | 0.29 |
| Popmax (GM) | 8.00 | 8.00 | 8.31 | 6.26 | 8.74 | 8.07 | 8.01 | 7.71 | 7.27 | 7.23 |
| Kkill (GM/h) | 0.19 | 0.06 | 0.14 | 0.49 | 0.12 | 0.13 | 0.34 | 0.25 | 0.45 | 0.91 |
| Hk | 1.30 | 3.67 | 17.20 | 2.32 | 1.70 | 1.28 | 1.79 | 2.61 | 2.18 | 11.07 |
| C50k (mg/L) | 2.50 | 2.07 | 0.30 | 2.01 | 2.40 | 2.04 | 3.05 | 2.67 | 3.36 | 1.71 |
| Initial Condition (GM) | 0.58 | 0.85 | 0.16 | 0.10 | 0.48 | 0.32 | 0.05 | 0.71 | 0.17 | 0.63 |
| Coefficient of determination for Observed-Predicted values for PK | 0.85 | 0.84 | 0.84 | 0.85 | 0.85 | 0.85 | 0.85 | 0.84 | 0.97 | 0.91 |
| Coefficient of determination for Observed-Predicted values for PD | 0.44 | 0.30 | 0.75 | 0.84 | 0.48 | 0.72 | 0.73 | 0.61 | 0.96 | 0.82 |
